# Spindle cell oncocytoma of the adenohypophysis in a woman: a case report and review of the literature

**DOI:** 10.1186/1752-1947-5-64

**Published:** 2011-02-14

**Authors:** M Mlika, H Azouz, I Chelly, I Ben Saïd, H Jemel, S Haouet, M Zitouna, N Kchir

**Affiliations:** 1 Department of Pathology, La Rabta Hospital, Bab Saadoun, Tunis 2037, Tunisia; 2 Department of Neurosurgery, La Rabta Hospital, Bab Saadoun, Tunis 2037, Tunisia

## Abstract

**Introduction:**

Spindle cell oncocytoma of the adenohypophysis is a rare tumour recently reported by Roncaroli *et al. *in 2002. This tumour is considered a grade I tumour by the World Health Organization.

**Case presentation:**

We describe what is, to the best of our knowledge, the 14th case of its kind in the literature. A 45-year-old African woman presented clinical and radiological findings related to a nonfunctioning pituitary adenoma. The diagnosis was made on the basis of histological and immunohistochemical findings.

**Conclusion:**

The purpose of this work is to report a rare pituitary tumour and to describe its histological and immunohistochemical features, which were characterized by the expression of thyroid transcription factor 1 antigen by tumour cells. This fact could support the theory of a possible common origin of these tumours in pituicytomas. In fact, thyroid transcription factor 1 is considered to be a specific marker of pituicytes.

## Introduction

Spindle cell oncocytoma (SCO) of the pituitary gland is a recently described entity which was recognized by the 2007 *WHO Classification of Brain Tumours *and considered a WHO grade I tumour [1]. It was initially described by Roncaroli *et al. *in 2002 [2], and only 14 cases have been reported in the literature. The histogenesis and prognosis of these tumours remain uncertain and need to be documented more thoroughly in the literature. Our aim is to report a new case of SCO and to describe its histological and immunohistochemical features supporting the theory of a possible common origin with pituicytoma [2].

## Case presentation

**Figure 1:**
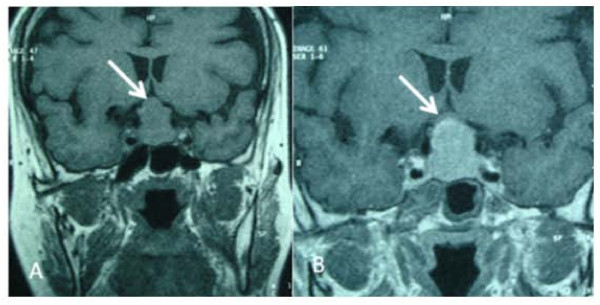
**(A) Coronal magnetic resonance imaging studies showing a sellar mass with suprasellar extension but no invasive growth (arrow)**. **(B) **T1-weighted image showing the enhancement of the mass (arrow).

We report the case of a 45-year-old African woman without a particular medical history who presented with intermittent decrease of visual acuity and headache. Cranial magnetic resonance imaging (MRI) revealed a solid adenohypophysis mass of 2 × 1.5 × 1 cm with suprasellar extension but no invasive growth. This mass showed contrast enhancing in T1-weighted MRI scans (Figure 1). Laboratory tests used to explore pituitary disorders showed normal levels of pituitary hormones, including follicle-stimulating hormone (FSH) (N > 20 IU/L), luteinizing hormone (LH) (N > 10 IU/L), prolactin (N < 20 μg/L), corticotropin and thyrotropin. The diagnosis of nonfunctioning pituitary macroadenoma was suspected, and the tumour was completely resected via transsphenoidal surgery. No adjuvant therapy was administered. Postoperatively, the patient developed panhypopituitarism which has been managed by hormone substitution. In fact, laboratory tests showed marked low levels of FSH (5 IU/L), LH (2 IU/L), prolactin (0.04 μg/L), corticotropin (10 nmol/L), thyrotropin (0.01 μU/mL) and somatotropin. Otherwise, currently there is neither clinical nor radiological evidence of a recurrent tumor after a three-month follow-up period.

**Figure 2:**
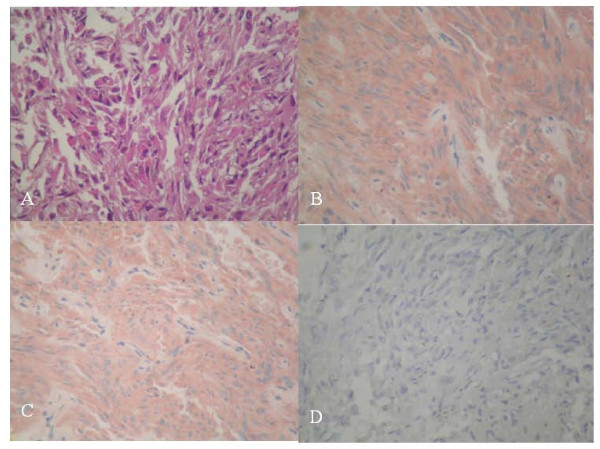
**(A) Histology and immunoprofile of spindle cell oncocytoma**. Spindle cell neoplasm with interlacing fascicles of spindled to epithelioid cells with eosinophilic and oncocytic cytoplasm (original magnification, ×400; hematoxylin and eosin stain). **(B) **Immunohistochemical study showed that most tumor cells coexpressed S-100 protein (original magnification, ×400; hematoxylin and eosin stain) and **(C) **vimentin and epithelial membrane antigen (original magnification, ×400; hematoxylin and eosin stain). **(D) **Tumor cells were negative with pituitary hormones.

Microscopic findings consisted of a solid spindle cell neoplasm with increased cellularity. Tumour cells were spindled to epithelioids organized in interlacing fascicles. The tumour cells had eosinophilic and oncocytic cytoplasm (Figure 2A). Nuclear atypia and pleomorphism were absent. Mitotic count was estimated to 1 per 10 high-power field. There were neither microvascular proliferations nor necrosis.

**Figure 3:**
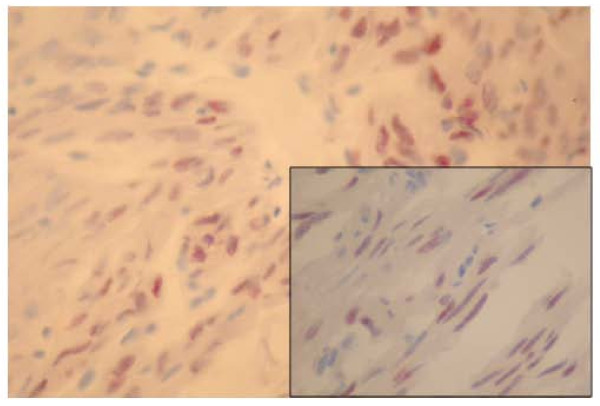
**Nuclear expression of thyroid transcription factor 1 by tumour cells (original magnification, ×200; hematoxylin and eosin stain)**. *Inset*: A higher-magnification image showing the nuclear expression (original magnification, ×400; hematoxylin and eosin stain).

An immunohistochemical study showed that most tumour cells expressed S-100 protein (Figure 2B). Vimentin and epithelial membrane antigen (EMA) were similarly expressed by tumor cells (Figure 2C). There was no staining either with low-molecular-weight cytokeratin or with anterior pituitary hormones, including somatotropin, corticotropin, thyrotropin, FSH, LH and prolactin (Figure 2D). Tumour cells expressed the thyroid transcription factor 1 (TTF-1) antigen (Figure 3). Glial fibrillary acidic protein (GFAP) and CD68 were not expressed. Therefore, the diagnoses of glial tumour and granular cell tumour were ruled out. In light of these histological and immunohistochemical findings, the diagnosis of SCO was strongly suspected. Ultrastructural examination showed neoplastic cells filled with mitochondria and well-formed desmosomes. These findings supported the diagnosis of SCO.

## Discussion

SCO is a rare tumour, with only 14 cases reported in the English-language literature (Table 1). In accord with the present case, it affects middle-aged and older adults of both sexes. It presents as a sellar tumour suspected to be a functionally inactive macroadenoma.

**Table 1:** Cases reported in the literature^a^

Year of publication	Reference	Number of reported cases	Sex ratio (M/F)	Mean age (yr)	Symptoms	Evolution
2002	Roncaroli *et al. *[2]	5 cases	3/2	61.6	Panhypopituitarism, visual defect	No recurrence (35.4 mo)
2005	Dahiya *et al. *[3]	2 cases	2 F	-	Panhypopituitarism	No recurrence
2005	Kloub *et al. *[4]	2 cases	1/1	73	-	Recurrence after 1 and 11 yr
2006	Vajtai *et al. *[5]	1 case	F	48	Adynamia, decrease of visual acuity	No recurrence
2009	Borota *et al. *[6]	1 case	F	-	-	Slow regrowth (30 mo)
2009	Demmsie *et al. *[7]	1 case	M	-	Visual blurring, weight loss	Recurrence after 9 mo
2009	Coiré *et al. *[8]	1 case	F	63	Visual defect	Recurrence after 5 mo

^a^M, male; F, female.

### Imaging findings

The radiological findings are nonspecific and do not differentiate these tumours from pituitary adenomas. In our case, they consisted of an enhanced mass of the sellar region.

### Histological and immunohistochemical features

Histological examination is the only means of diagnosis [2-4]. This tumour is typically composed of interlacing fascicles of spindled to epithelioid cells with oncocytic cytoplasm. Mild to moderate nuclear atypia and even focal marked pleomorphism may be seen. The immunoprofile of these tumours is characterized by simultaneous positivity for S-100 protein, vimentin and EMA. Ultrastructurally, the neoplastic cells contain numerous mitochondria with lamellar cristae. The neoplastic cells are linked by intermediate junctions and desmosomes [1,5].

### Pathogenesis

These histological, immunohistochemical and fine structural features lead most authors to postulate a possible derivation of these tumours from folliculostellate cells. Very little is known about the functioning of the folliculostellate cells. Some authors have reported that these cells are implicated in long-distance communication in the anterior pituitary gland [6-8]. These cells are known to coexpress the S-100 protein, vimentin and galectin 3. These findings are shared by the SCO, but in our case tumour cells also expressed TTF-1. Lee *et al. *[9] described the expression of TTF-1 in eight cases of SCO. They reported that TTF-1 is generally expressed in fetal neurohypophysis. According to these findings, this marker could be specific to human pituicytes. The positivity of TTF-1 in our observation with these eight reported cases should lead to further research that could have implications for the classification of these rare sellar neoplasms and may indicate a similar origin of SCO and pituicytoma [9].

### Differential diagnoses

The diagnosis of these tumours may be challenging. In fact, they should be distinguished from meningioma in its oncocytic variant, granular cell tumour, pituicytoma, oncocytic neoplasm arising from developmental salivary gland remnants and oncocytic variant of a pituitary adenoma [2-4]. Rarely, they should be distinguished from sellar schwannoma, but this tumour is very rare in that location [10]. Most meningiomas that are located within the cranial cavity occur over the cerebral convexities, but other common sites include para- and suprasellar regions. The radiological findings may be challenging when showing an enhancing mass without particular characteristics. The pathological findings and immunohistochemical features show a similar expression of EMA and vimentin, but S-100 protein is rarely expressed in meningiomas. Moreover, in opposition to SCO, tumour cells in meningiomas are filled with intermediate filaments and desmosomal intercellular junctions in ultrastructural examination [2]. Granular cell tumours and pituicytomas tend to develop in the posterior pituitary gland rather than in the adenohypophysis. These tumours are thought to originate from pituicytes. Granular cell tumors are characterized by a granular cytoplasm which can be observed in SCO, but, in opposition to the SCO, granular cell tumour shows a strong expression of CD68. Besides, the cytoplasm of the granular cells is filled with phagolysosomes, and there are no mitochondria. The distinction from pituicytoma relies on the evidence of oncocytic change in SCO rather than GFAP staining patterns alone. Oncocytic neoplasms originating from salivary gland remnants express epithelial markers and lack S-100 protein and EMA positivity [3,4]. The distinction of SCO from an oncocytic variant of a pituitary adenoma is based on the expression of neurosecretory markers synaptophysin and chromogranin by the adenoma [5]. The differences in immunohistochemical profile between these tumours are illustrated in Table 2. The treatment of these tumours is based on surgical resection. Postoperative complications consist mainly of hypopituitarism as in the case of our patient. This complication is due to the difficulty of this surgery, which needs accurate management that is not always possible in the sellar region. A consensual protocol has not been assessed because of the complex issue of these tumours and the lack of large series. In fact, among the 14 cases reported in the literature, eight patients had a benign clinical course and six experienced recurrence despite adjuvant treatment in two cases [6].

**Table 2:** Immunohistochemical findings in spindle cell oncocytoma and the main differential diagnoses^a^

Diagnoses	Immunohistochemical markers
Spindle cell oncocytoma	S-100 protein, vimentin and EMA are expressed
Oncocytic variant of meningioma	EMA is expressed, vimentin is expressed and S-100 protein is negative
Granular cell tumour	CD68 is expressed
Pituicytoma	GFAP is expressed
Oncocytic neoplasm originating from salivary gland remnants	Epithelial markers are expressed, S-100 protein and EMA are negative
Oncocytic variant of a pituitary adenoma	Synaptophysin and chromogranin are expressed

^a^EMA, epithelial membrane antigen; GFAP, glial fibrillary acidic protein.

## Conclusion

SCOs of the pituitary gland are rare tumours whose pathogenesis and management remain debated because of the few numbers of reported cases. These tumours are considered to have a good prognosis despite the early recurrences reported in some cases [8,9]. Additional clinical follow-up is needed to assess the prognostic features. In our case, the period of follow-up was too short, so we can only speculate whether such a tumour is benign.

## Competing interests

The authors declare that they have no competing interests.

## Consent

Written, informed consent was obtained from the patient for publication of this case report and accompanying images. A copy of the written consent is available for review by the Editor-in-Chief of this journal.

## Authors' contributions

MM conceived of, coordinated with other coauthors and drafted and revised the manuscript. HH, IC, IBS, SH, HJ, MZ and NK participated by acquisition and analysis of literature data and helped to draft the manuscript. All authors read and approved the final manuscript.
